# Study on tunnel ventilation and pollutant diffusion mechanism during construction period

**DOI:** 10.1371/journal.pone.0322984

**Published:** 2025-05-12

**Authors:** Xiaoke Chang, Jianxi Ren, Rong Yang, Yan Tong

**Affiliations:** 1 School of Architecture and Civil Engineering, Xi’an University of Science and Technology, Xi’an, Shaanxi, China; 2 College of Civil Engineering, Shaanxi Polytechnic Institute, Xi’an, Shaanxi, China; 3 Geological Survey and Engineering Institute, Northwest Engineering Corporation Limited, Xi’an, Shaanxi, China; Southwest Jiaotong University, CHINA

## Abstract

Ventilation technology is an important means to ensure effective control of pollutant concentration and safe production during tunnel drilling and blasting construction. This study combines theoretical derivation, numerical simulation, and mathematical statistics to explore the extraction of flow field distribution characteristics and pollutant transport and diffusion mechanisms in tunnels. The research results indicate that the instability and turbulence effects of fluids work together to form a vortex zone near the tunnel working face. Fluid instability refers to the tendency of fluids to undergo changes under the influence of tunnel sidewalls or airflow in ducts. Turbulence effect is caused by the chaotic and irregular flow of fluids, leading to fluid mixing and rotation. The complex flow field changes inside the tunnel result in the retention of pollutants generated during construction in specific zone. The main reasons for the formation of pollutant stagnant zones are the bypass effect, low-velocity regions, and vortex of fluid. The emission process of pollutants can be divided into two stages: extraction and dilution. The dilution effect of pollutants is inversely proportional to the distance between the air duct and the working face, and the extraction amount is directly proportional to the airflow of the fan. The shorter distance allows fresh air to directly reach high concentration pollutant zone from the air duct, accelerating the mixing and dilution process. A larger airflow can provide stronger power and carry more pollutants out of the tunnel. The improved Technique for Order Preference by Similarity to an Ideal Solution method can optimize the layout of ventilation parameters and improve ventilation conditions. Finally, an empirical calculation formula for air supply volume is derived through in - depth research and data analysis. This formula takes into account multiple factors related to the tunnel structure, pollutant generation, and ventilation requirements. This empirical formula provides a scientific basis for the selection of ventilation fans in the construction preparation stage. Construction planners can accurately calculate the required air supply volume according to the specific situation of the tunnel, and then select the appropriate ventilation fan, which can not only ensure the ventilation effect but also save energy and reduce costs.

## 1. Introduction

The design and operation of ventilation systems during tunnel construction are of great significance for ensuring worker safety, improving air quality, reducing environmental pollution, and enhancing construction efficiency. Due to the complex internal environment of the tunnel, the ventilation effect is affected by various factors such as obstacles, ventilation techniques, and airflow distribution. Therefore, studying the optimization design and operational strategies of ventilation systems in construction tunnels has important theoretical and practical significance.

The main function of tunnel ventilation system is to provide sufficient fresh air, dilute and discharge harmful gases and dust, and maintain the air quality inside the tunnel. According to research data, ventilation system failure is one of the main factors leading to disasters during tunnel construction. The cost of ventilation usually accounts for about 30% to 50% of the total investment in tunnel engineering (the cost of ventilation is directly proportional to the third power of the tunnel excavation length) [1]. Studying the ventilation system of tunnels during the construction period is a necessary measure to save energy and ensure safety.

The parameters of ventilation airflow field are the key to tunnel ventilation design. Research has found that parameters such as ventilation velocity, air volume, air direction, and air pressure have a significant impact on the air quality inside tunnels [2–5]. Appropriate air velocity can effectively dilute and discharge harmful gases and dust. Tascón and Aguado [6] studied the influencing parameters of dust explosion discharge through simulation and pointed out that air velocity is one of the key factors. Excessive or insufficient air velocity can affect ventilation effectiveness and directly lead to the distribution and concentration of harmful gases in tunnels [2]. Appropriate air volume can save fan energy consumption and reduce resource consumption while ensuring that the air quality inside the tunnel meets standards [7]. The change in air direction can also affect the diffusion path of harmful substances, thereby affecting the air quality inside the tunnel [8]. The reasonable distribution of air pressure can ensure the uniformity and effectiveness of airflow [9].

By studying ventilation parameters, the system layout on site can be optimized. The practical application of ventilation systems involves multiple aspects, including the selection, layout, and operation strategies of ventilation equipment. Reasonable ventilation system design can significantly improve the safety and air quality of construction tunnels [9–14]. Select appropriate ventilation equipment based on the length of the tunnel, terrain, and construction conditions. Axial flow fans are suitable for ventilation control in long tunnels, while jet fans are suitable for ventilation control in short tunnels and local zone [9]. Adjust the operation strategy of ventilation equipment according to the actual situation inside the tunnel, such as adjusting air velocity, air volume, and air direction. Intelligent control systems can automatically adjust based on real-time monitoring data to improve ventilation efficiency [15]. Reasonable equipment layout can improve ventilation efficiency and reduce energy consumption. The arrangement of fans should ensure even distribution of airflow, ensuring the uniformity and effectiveness of the airflow [11].

Carbon monoxide (CO) is one of the main harmful gases generated during tunnel construction. CO gas is not only harmful to human health, but its flammability and explosiveness may also cause serious safety accidents [16–20]. Therefore, studying the emission patterns and control methods of CO gas during tunnel ventilation has important practical significance. Research has found that a reasonable ventilation strategy can effectively reduce the concentration of CO gas in the tunnel after blasting and minimize its impact on the environment. By adjusting the ventilation velocity and the arrangement of ventilation openings, the air quality inside the tunnel can be significantly improved [21]. Different ventilation strategies have a significant impact on the diffusion of harmful gases. By optimizing the layout of the ventilation system and adjusting the ventilation velocity, the concentration of harmful gases can be effectively reduced [19]. In addition, for the harmful gases such as CO generated during vertical shaft construction and the optimization of ventilation system layout, numerical simulation technology can also be used for research to obtain the optimal layout plan [20]. The diffusion rate of CO gas in high-altitude tunnels is slow and the concentration distribution is uneven. In addition, optimizing the ventilation system can significantly improve the diffusion effect of CO gas and reduce the CO concentration in high concentration zone [16]. The research on ventilation engineering provides scientific basis for the optimization design of ventilation systems, and improves the safety and efficiency of tunnel and shaft construction.

Computational Fluid Dynamics (CFD) technology is widely used in the simulation and optimization of tunnel ventilation systems. Through CFD simulation, the distribution of airflow, diffusion paths of pollutants, and concentration changes in tunnels can be analyzed in detail, providing scientific basis for the design of ventilation systems [16–19,22]. Establishing an accurate CFD model is the foundation of simulation. The model should include parameters such as the geometric structure of the tunnel, layout of ventilation equipment, air velocity and pressure [22]. Reasonable boundary conditions can improve the accuracy of simulation. Common boundary conditions include inlet air velocity, outlet pressure, and wall conditions. Verify the accuracy of simulation results through on-site experimental data to ensure the reliability and applicability of the model. Sota et al. [23] optimized the ventilation system of a highway tunnel. Through CFD simulation, the effects of different ventilation schemes were analyzed, and the optimal ventilation scheme was ultimately selected. The results indicate that the optimized ventilation system significantly improves the air quality inside the tunnel and reduces the concentration of harmful gases. Pang et al. [24] studied the ventilation system of underground metal mine tunnels. The influence of ventilation velocity and air volume on dust concentration was analyzed through CFD simulation. Reasonable ventilation parameters can effectively reduce dust concentration and improve the air quality inside tunnels. Yang et al. [25] used CFD technology to optimize the ventilation flow field of long tunnels. By simulating the flow field distribution under different ventilation schemes, they found the optimal ventilation layout to minimize the concentration of pollutants in the tunnel. These studies have demonstrated the potential application of CFD technology in tunnel ventilation design.

Computational Fluid Dynamics (CFD) is the analysis of systems containing physical phenomena such as fluid flow and heat conduction through numerical calculations and image displays. Its advantages are reflected in: 1. The CFD method only requires the establishment of corresponding mathematical models and grids on the computer to simulate fluid flow under different working conditions, greatly reducing experimental costs and quickly evaluating and optimizing different design schemes. Avoiding the process of repeatedly manufacturing and testing physical models in traditional design significantly shortens the research cycle. 2. CFD simulation can provide detailed information about any location in a fluid system, including the distribution of physical quantities such as velocity, pressure, temperature, concentration, etc. And it can clearly observe the occurrence and development process of fluid vortex, separation, mixing and other phenomena, which helps to deepen the understanding of the physical mechanism of fluid flow. 3. CFD numerical simulation methods can also avoid many dangerous scenarios. For example, harmful gases such as CO in tunnels cannot be avoided from leaking through experimental research or on-site measurement, posing a threat to the safety and health of researchers.

Effective ventilation is a key factor in ensuring the safety and health of construction personnel and improving construction efficiency. CFD, as a powerful numerical simulation tool, has an irreplaceable necessity in studying ventilation during tunnel blasting construction. Compared with the limitations of on-site measurements and traditional experimental methods, CFD methods have the advantages of comprehensive flow field information acquisition, flexible working condition simulation, high cost-effectiveness, strong repeatability, and reliability. By evaluating and optimizing different schemes, CFD can provide more scientific and reasonable ventilation design solutions for practical engineering. CFD analysis of pollutant diffusion patterns and prediction of pollutant concentrations at different locations within the tunnel provide a basis for developing effective protective measures and ensuring the health of construction workers. Therefore, it is necessary to use CFD method in the study of ventilation during tunnel blasting construction, and it has broad application prospects.

The environmental impact and sustainability of tunnel ventilation systems have been a hot research topic in recent years. Research has found that a well-designed ventilation system can reduce energy consumption, minimize environmental impact, and improve the sustainability of tunnel construction. Optimizing ventilation parameters and equipment layout can significantly reduce energy consumption and lower operating costs [26]. A reasonable ventilation system can reduce the emission of harmful gases and dust, and protect the environment [15]. Through intelligent control technology, intelligent management of ventilation systems is achieved. By monitoring and predicting air quality in real-time, demand controlled ventilation is achieved, effectively reducing energy consumption and environmental pollution [15,26]. On demand air supply can improve the sustainability of tunnel construction [27].

In summary, significant progress has been made in the research of ventilation in construction tunnels in recent years. Research has shown that a reasonable ventilation strategy can effectively reduce the concentration of CO gas in tunnels, improve air quality, and enhance construction safety [28–30]. However, due to the blocking effect of airflow vortex and the influence of low-velocity airflow, there are many harmful gases accumulating inside the tunnel. The mechanism by which airflow affects the discharge of pollutants in the retention zone is not yet clear, resulting in significant estimation errors in the required air volume. Accurately estimating and controlling the required air volume can effectively reduce harmful gas emissions, minimize pollution to the surrounding environment, and protect air quality and ecological balance. And accurate estimation of air demand can optimize the operation of ventilation systems, reduce power consumption, and lower overall project costs. Thus the engineering can reduce carbon emissions and support the development of a low-carbon economy. This study comprehensively considers the interaction between airflow and pollutants, and adopts numerical simulation and theoretical derivation methods to study the influence of different parameters on the spatiotemporal distribution of pollutants. By fully considering the effects of airflow convection and diffusion, a new empirical formula for calculating supply air volume is derived. The results of this study can supplement the research direction of pollutant control, play an important role in environmental protection, resource utilization, and provide strong support for the planning and management of tunnel construction ventilation systems.

## 2. Numerical method and model analysis

### 2.1 Numerical method

The assumptions used for numerical simulation are based on the literature [8,17,18,20]:

The pollutants generated during tunnel drilling and blasting mainly include dust, harmful gases (such as carbon monoxide, nitrogen oxides, sulfur dioxide, etc.), but the proportion of different pollutants will be significantly affected by multiple factors. The proportion of harmful gases in pollutants is approximately 20% -40%. The main components are carbon monoxide and nitrogen-containing oxides. The composition of the components is related to the type and quantity of explosives, blasting conditions, and geological conditions.

When analyzing the emission pattern of harmful gases in tunnel blasting, most scholars choose CO (carbon monoxide) as the tracking gas, mainly based on the following reasons: 1. The production of CO is usually more significant, which makes it more concentrated in harmful gases and easier to detect and track. 2. CO is difficult to diffuse and easily accumulates in tunnels to form high concentration zone, posing a serious threat to the safety of construction workers. 3. The chemical properties of CO are relatively stable. Under the environmental conditions inside the tunnel, it is not easily consumed or converted by chemical reactions with other substances. This means that during the propagation and diffusion of CO in tunnels, its concentration changes are mainly influenced by physical factors such as ventilation and diffusion, making it easier for researchers to analyze the emission patterns of harmful gases by monitoring its concentration changes.

Therefore, this study uses the total amount of CO gas converted from harmful gases generated by blasting as the blasting pollutant to study the migration law of pollutants. And it is assumed that CO is uniformly distributed within the throwing range under the initial conditions after blasting.

Based on the above assumptions, the mathematical model was analyzed and the unsteady Navier-Stokes equations were used as the governing equation[4–6, 17].

Continuity equation:


$$\partial \rho /\partial t+\partial (\rho u_{i})/\partial x_{i}=0$$ (1)

Momentum conservation equation:


$$\partial u_{i}/\partial t+u_{i}\partial u_{i}/\partial x_{i}=g_{i}-\partial p/(\partial \rho x_{i})+\partial^{2}\mu u_{i}/(\rho \partial x_{i}\partial x_{i})$$ (2)

Energy conservation equation:


$$\partial (\rho T)/\partial t+\partial (\rho u_{j}T)/\partial x_{j}=\partial (\partial (\mu_{t}T)/\partial (\sigma Tx_{j}))/\partial x_{j}+(C_{pv}-C_{pa}partial(\mu_{t}\omega )\partial T/C_{p}\partial (\sigma Tx_{j})\partial x_{j}$$ (3)

Species transport equation:


$$\partial (\rho \omega )/\partial t+\partial (\rho u_{j}\omega )/\partial x_{j}=\partial (\partial u_{t}\omega /\partial x_{j})/\partial x_{j}$$ (4)

Considering the influence of vortex on turbulence, the computational accuracy of complex flows has been improved. Renormalization-group (RNG) k−ε turbulence model is introduced to solve the diffusion problem of CO gas in tunnel. The equations are as follows [17,30,31]:


$$\partial (\rho k)/\partial t+\partial (\rho ku_{j})/\partial x_{j}=\partial ((\mu +\mu_{t}/\partial x_{j}cdot\partial k/\partial x_{j})/\partial x_{j}+G_{k}-\rho \varepsilon +S_{k}$$ (5)

$$\partial (\rho \varepsilon )/\partial t+\partial (\rho \varepsilon u_{i})/\partial x_{i}=\partial ((\mu +\mu_{t}/\sigma_{\varepsilon }partial\varepsilon /\partial x_{j})/\partial x_{j}+c_{1\varepsilon }G_{k}\varepsilon /k-c_{2\varepsilon }\rho \varepsilon^{2}/k-R_{\varepsilon }+S_{\varepsilon }$$ (6)

Where, k is the turbulent kinetic energy; ε is the turbulent kinetic energy dissipation rate; $G_{k}$ is the turbulence kinetic energy produced by the laminar velocity gradient; μ respectively denote the viscosity; S denote the source terms; $R_{\varepsilon }$ is an additional term of ε equation; $c_{\mu }$, $c_{\varepsilon }$ and σ are the model constants.

Assuming that the velocity distribution, turbulent kinetic energy, and energy ratio at the inlet boundary of the tunnel duct are uniformly distributed [4–6]. The air velocity uin in the x-direction at the inlet of the air duct is determined by the cross-sectional area A of the air duct and the ventilation volume Q, $u_{in}=Q/A$ 。The y-direction and z-direction velocities are both 0. Turbulent kinetic energy, taken as 0.005.

The exit boundary is the tunnel exit, using uniform flow boundary conditions. The gradient of each export variable in the yz plane is set to 0. The relative pressure at the exit of the tunnel is set to 0, $P=P_{out}=0$.

The side walls, bottom surfaces, and working faces of the tunnel are all set as fixed wall boundary conditions and treated according to the law of fixed walls. The node at the fixed wall adopts no slip condition, and the pressure gradient perpendicular to the wall is 0. When given initial conditions, based on on-site experience, measured results, and relevant literature, the migration equation of harmful gases over time is [6]:


$$\frac{\partial }{\partial t}\left(\rho Y_{m}\right)+\frac{\partial }{\partial x_{j}}\left(\rho v_{j}Y_{m}\right)=\frac{\partial }{\partial x_{j}}\left(\frac{\mu_{e}}{\sigma_{Y}}\frac{\partial Y_{m}}{\partial x_{j}}\right)$$ (7)

### 2.2 Discussion of grid independence

**Fig 1:**
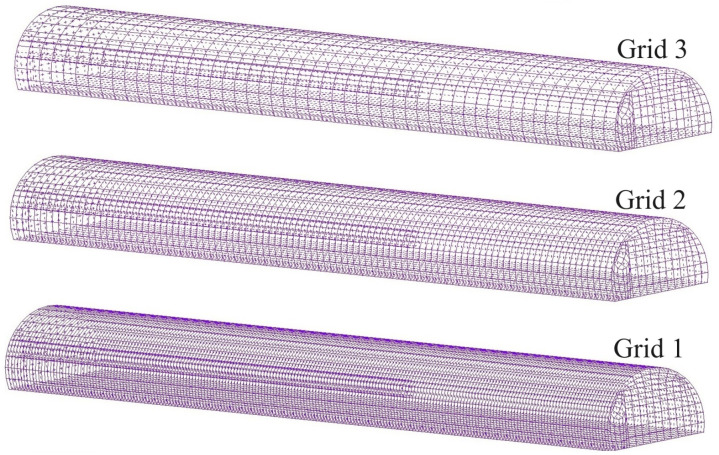
Grid division of calculated model.

In order to ensure the accuracy of numerical simulation results, it is necessary to explore the independence of the grid [32]. Fig 1 shows the schematic diagram of the tunnel model meshing.

This study uses the Grid Convergence Index (GCI) method to verify the independence of the grid[33]. Three 100m-long tunnel models with different grid scales were established, including 274303, 137154, and 68623 elements. The grid convergence index can be obtained by the following equation:


$$GCI_{i+1,i}^{fine}=F_{s}e_{a}^{i+1}/(r_{i+1}^{p}-1times100\;$$ (8)

Where $F_{s}$ is the safety factor, taken as 1.25; e is the relative difference; $r_{i+1,i}$ is the grid refinement factor; p is the convergence accuracy.

The convergence ratio R is calculated by:


$$R=(f_{2}-f_{1})/(f_{3}-f_{2})$$ (9)

Where $f_{i}$ is the the average air velocity.

Asymptotic domain index β is calculated by:


$$\beta =r_{21}^{p}GCI_{21}^{fine}/GCI_{32}^{fine}$$ (10)

**Fig 2:**
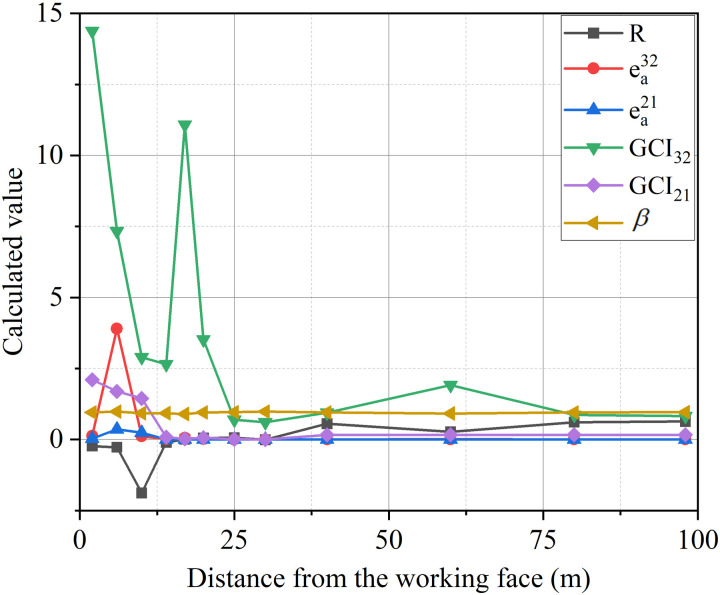
Calculated results of model.

Fig 2 shows the evaluation results of the independence of different grid models using the GCI method. When 0<X < 14 m, R < 0. The numerical solution for air velocity distribution within this interval is in a state of vibration and the results tend to converge. When X > 14 m, 0<R < 1. The numerical solution of air velocity distribution within this interval is in a monotonic convergence state. Therefore, it is believed that the numerical calculation within this interval is less affected by the disturbance of grid density. The results of $e_{a}^{i+1,i}$, GCI and β with the gradual refinement of the grid, indicating influence of grid density on numerical calculation results tends to decrease at the same time. Therefore, the density of grid 2 has basically met the calculation requirements. In order to simulate the flow field near the working face more precisely, the grid 1 is used for analysis in this zone. The remaining zone of the tunnel are divided into grid 2 density grids.

### 2.3 Model validation

**Fig 3:**
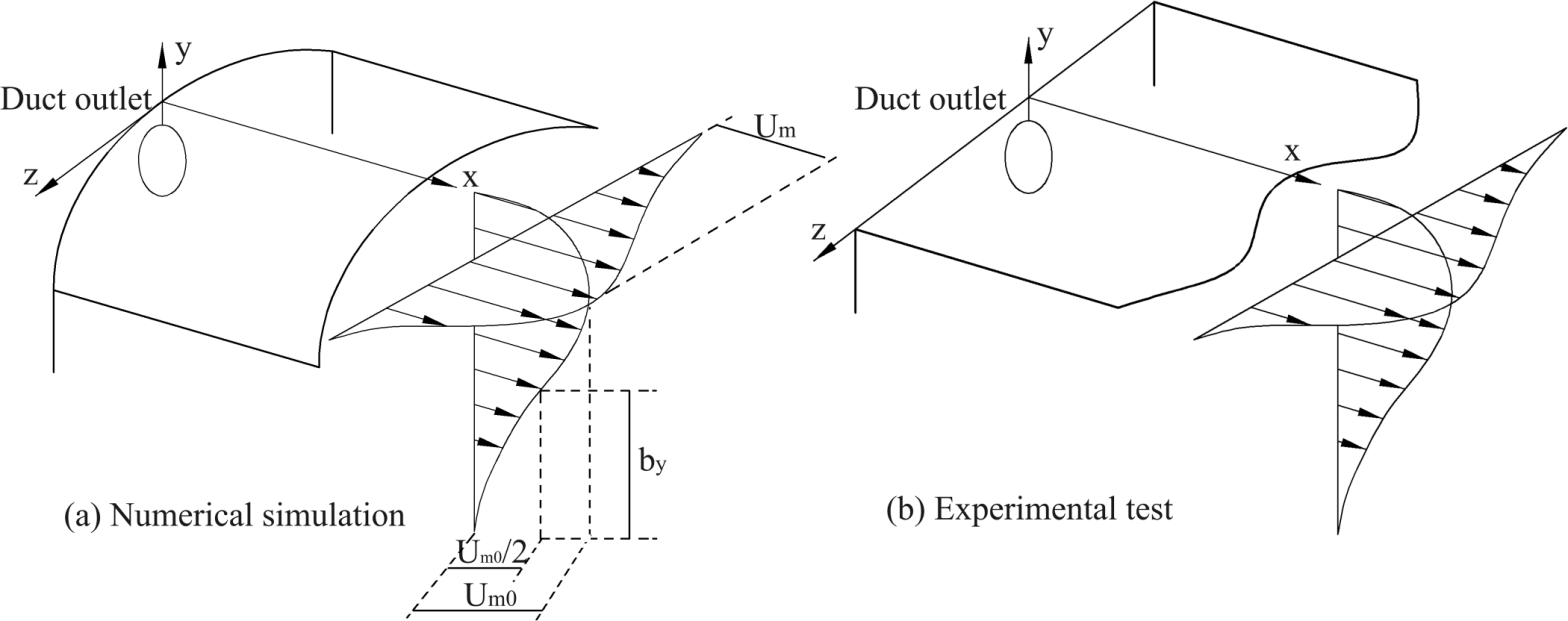
Three-dimensional wall jets sketch.

This study validated the accuracy of the mathematical model using the experimental results of circular wall jet conducted by Rajaratnam and Pani [34]. The symbols for the three-dimensional circular wall jet used for numerical simulation and experiments are shown in Fig 3.

**Fig 4:**
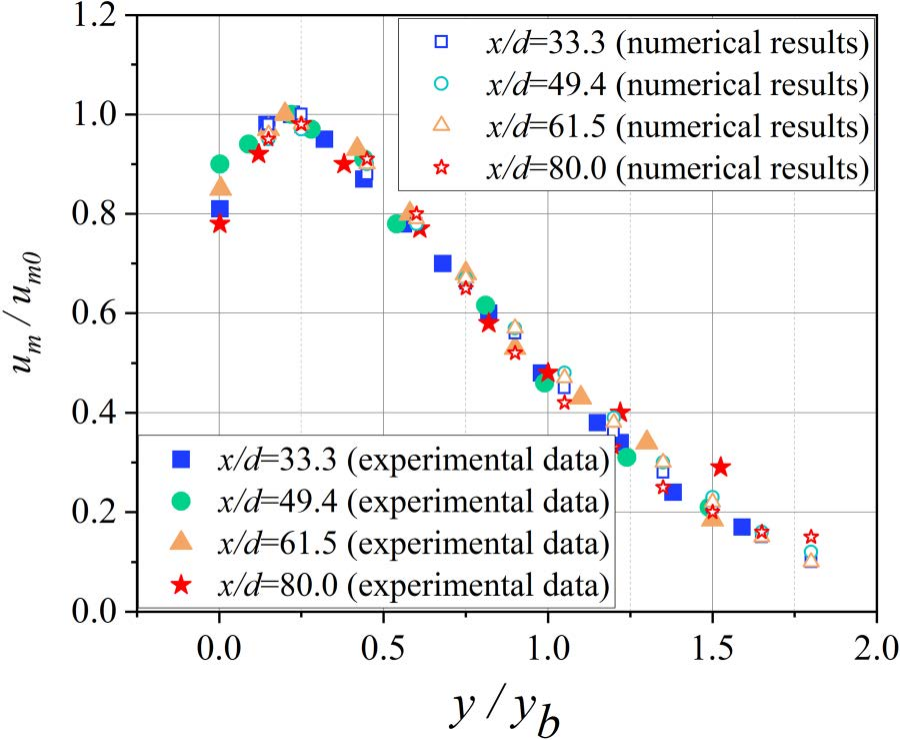
Non dimensional velocity distribution in the radial decay region of the central plane.

**Fig 5:**
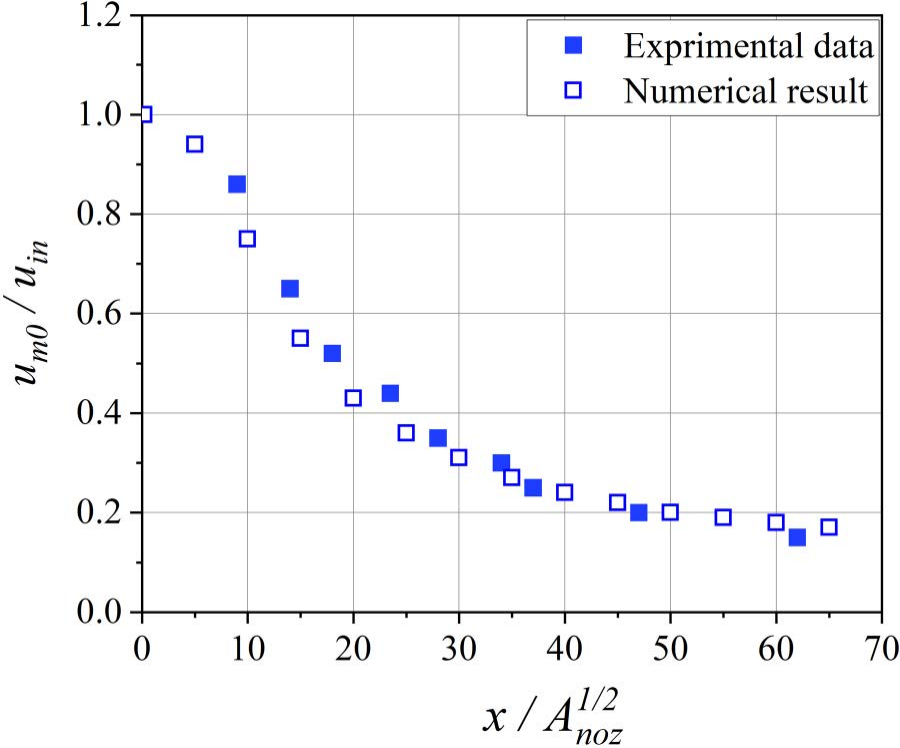
Non dimensional longitudinal maximum velocity distribution.

Figs 4-5 show the comparison between simulated and measured airflow velocity values. The calculation results show that both the dimensionless velocity distribution ($u_{m}/u_{m0}$) and the dimensionless longitudinal maximum velocity distribution ($u_{m0}/u_{in}$) can be well matched with the experimental results. Therefore, the model used in this study can accurately simulate the ventilation conditions inside the tunnel.

### 2.4 Physical model and boundary conditions

The calculation example adopts the transportation engineering of Lianghekou hydropower station. The ventilation model of the construction tunnel was established by ADINA software in a 1:1 ratio. The structure of a single head tunnel ventilation system is relatively simple, consisting of duct and tunnel. Extracted ventilation is a mechanical ventilation method used for underground tunnels. It uses ventilation fans installed outside the tunnel to transport polluted air to the outside of the tunnel through ventilation ducts, and fresh air flows through the tunnel channel to the working face. The model size is 500m × 11m × 7.3m, and the tunnel section area is 67m^2^. The diameter of the tunnel air duct is 1.8m. The distance between the air duct outlet and the working face is 15m. The CO gas observed by the model is calculated based on the concentration values of pollutants converted into CO according to specifications. According to relevant standards, the maximum allowable concentration of CO during tunnel construction is 30mg/m^3^. In special circumstances, the worker must enter the tunnel at a rate of 100mg/m^3^, but the operation time must not exceed 30min [35].

### 2.5 Ethics statements

This research did not involve human or animal subjects. All data used in this study were obtained from publicly available sources, and we have followed all relevant laws and regulations regarding data collection and usage. We have also ensured that all intellectual property rights have been respected. Any copyrighted materials used in this research were properly cited and used within the scope of fair use.

## 3. Results and discussion

### 3.1 Analysis of tunnel flow field characteristics and pollutant transport laws

**Fig 6:**
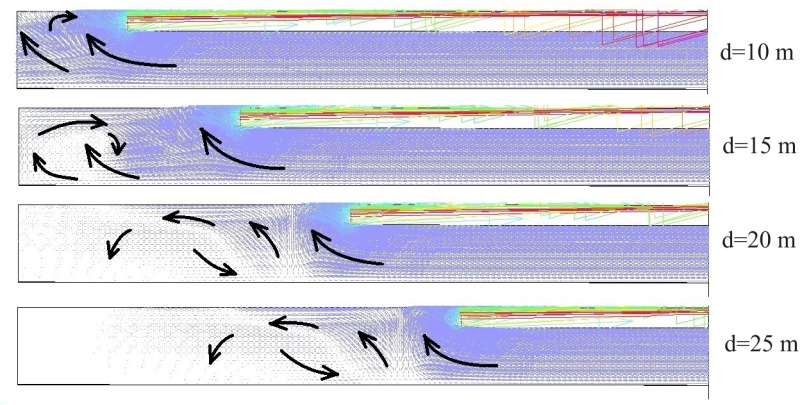
Airflow field analysis diagram of extracted ventilation under different conditions.

**Fig 7:**
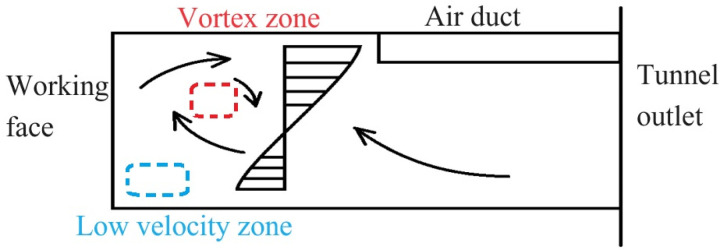
Flow velocity zoning diagram.

The transport and diffusion process of the CO gas is based on fluid motion. Adopting the extracted ventilation mode, the airflow inside the tunnel is sucked out of the tunnel space through air ducts. Pollutants are also discharged from the tunnel through air ducts. The air velocity vector diagram of the tunnel were obtained based on the simulation results. The distribution pattern of airflow is basically similar when the distance between the outlet of the air duct and the working face is different. The distribution of airflow field in tunnel was shown in Figs 6-7.

The distribution of airflow in the zone between the duct outlet and the working face is complex. The turning point where the airflow tends to stabilize is near the duct outlet. Under the action of air pressure, the airflow pushes the gas near the working face towards the duct. Fresh air enters from the tunnel outlet and flows towards the working face. Due to the influence of the jet flow and the working face wall, there is vortex distribution near the tunnel working face. As the distance between the duct outlet and the working face increases, the vortex distribution zone gradually increases. And the distribution range of low velocity airflow zone is gradually expanding. Vortex and low velocity airflow zone become blind spots for ventilation.

The reasons for this phenomenon are: 1. Changes in tunnel morphology and roughness of tunnel walls cause turbulence in the airflow, which is an important factor in the formation of vortex; 2. The velocity and direction of the fluid undergo significant changes with the shape of the tunnel. This velocity gradient (shear layer) leads to fluid instability. The instability of the shear layer of the fluid triggers the generation of vortex; 3. The extraction ventilation mode causes the airflow inside the tunnel to be in a negative pressure state. The negative pressure effect can cause uneven airflow inside the tunnel, resulting in large-scale turbulent flow patterns; 4. The airflow inside the tunnel is a mixed flow state with a certain degree of turbulence. Turbulence leads to random mixing and redistribution of airflow. Turbulent energy generates vortex structures during dissipation, ultimately forming vortex.

**Fig 8:**
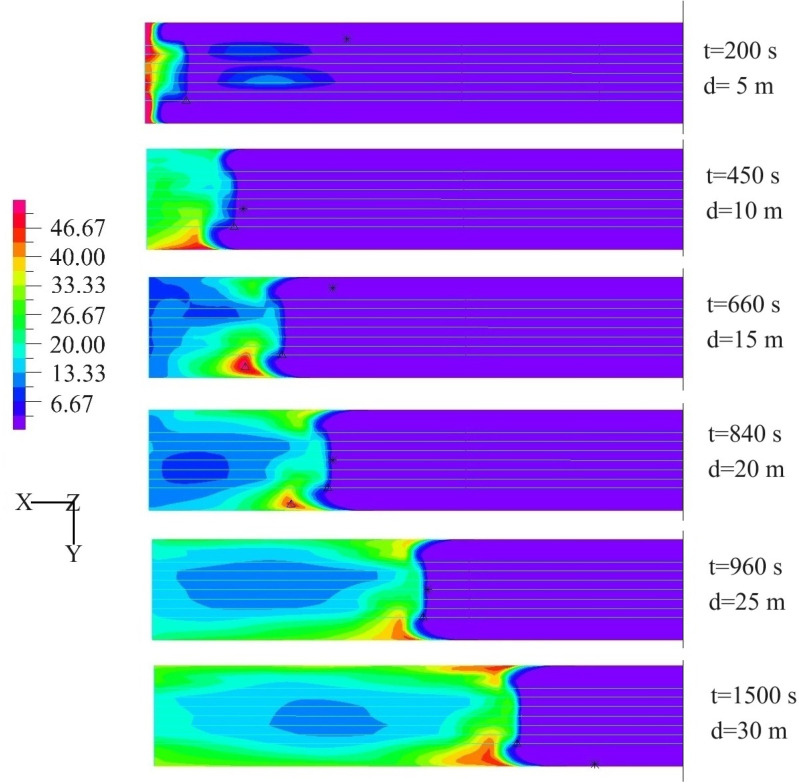
Harmful gas retention under different conditions.

Fig 8 shows the retention of CO gas in the tunnel. The process of CO being discharged from the tunnel can be clearly obtained.

In the extracted ventilation mode, the contaminated gas within the tunnel gradually migrates towards the working face. During this migration process, the spatial distribution range of pollutants progressively contracts. As the ventilation duration extends, a stagnant region of pollutant emission emerges in the lower area adjacent to the working face. In this stagnant region of pollutant gas emission, the harmful gases do not vanish with the increase of ventilation time; instead, their concentration values consistently decline. Consequently, in the extracted ventilation mode, the pollutant discharge process inside the tunnel can be categorized into two distinct phases: extraction and dilution. In the initial stage of ventilation, CO gas is predominantly removed through the air - duct system. In the subsequent stage of ventilation, the CO gas is mainly diluted as a result of the influence of the internal flow - field distribution within the tunnel.

From Fig 8, it can be seen that when the duct outlet is close to the working face (d = 5m), the stagnant zone is mainly concentrated near the working face. The overall range of the stagnant zone is relatively small, and its shape is roughly circular or elliptical. Due to the strong suction effect of ventilation, harmful gases are difficult to diffuse to distant places, and the boundaries of the stagnant zone are relatively clear. In the stagnant zone, the CO concentration is relatively high (55mg/m^3^). This is because harmful gases accumulate in large quantities near the working face in a short period of time, and the ventilation system has limited pumping capacity, resulting in a rapid increase in concentration.

As the distance between the duct outlet and the working face gradually increases (d = 20m), the stagnant zone begins to move towards the duct outlet direction. This is because the suction effect of the ventilation system gradually weakens, and harmful gases are less constrained during the diffusion process, starting to spread further away. At the same time, they are affected by the suction of the air duct and gradually gather near the duct outlet. The range of the stagnant zone gradually expands and its shape becomes more irregular. Due to the influence of various factors such as airflow and tunnel walls during the diffusion process, harmful gas is no longer limited to a small zone near the working face, but extends along the tunnel towards the direction of the duct outlet. The boundary of the stagnant zone becomes blurred, and the mixing zone with the surrounding air increases. The overall concentration of CO gas in the stagnant zone has decreased (45mg/m^3^), but the distribution is more uneven.

When the distance between the duct outlet and the working face is far (d = 30m), the stagnant zone form multiple dispersed zone in the tunnel. On the one hand, there is still a small amount of CO remaining near the working face. The reason is that the ventilation system has a relatively small suction effect in this zone; On the other hand, a large stagnant zone is formed near the duct outlet, and harmful gas retention also occurs in some zone in the middle of the tunnel. This is because the airflow becomes turbulent during long-distance transmission, causing harmful gases to be unable to be smoothly extracted. The concentration of harmful gases in the stagnant zone is generally low. Due to the large diffusion range of harmful gases, they are fully mixed with the surrounding air. There are high CO concentration points in local zone (42mg/m^3^), which poses greater difficulties for ventilation management.

The reasons for this phenomenon are: 1. The extracted ventilation mode causes the airflow inside the tunnel to be in a negative pressure state. The negative pressure effect cause uneven airflow inside the tunnel. Formation of vortex distribution zone and low flow velocity zone near the working face; 2. The vortex zone blocks the airflow path, causing pollutants to accumulate at the center of the vortex and difficult to disperse. The decrease in air velocity in local zone leads to slow diffusion of pollutants; 3. When the distance between the duct outlet and the working face is large, air is prone to flow towards the ventilation equipment through the shortest path. The fresh air supply near the working face is insufficient, forming a stagnant zone.

### 3.2 The impact of different factors on pollutant emissions

This study established multiple different working conditions, considering various factors to explore the emission mechanism of pollutants under the extracted ventilation mode. According to flow field characteristic, the stagnant zone is easily formed near the working face. The vortex in the stagnation zone and low flow velocity zone have a blocking effect on the discharge of harmful gases. The stagnation zone is selected as the research object to observe the variation process of harmful gas over time.

**Fig 9:**
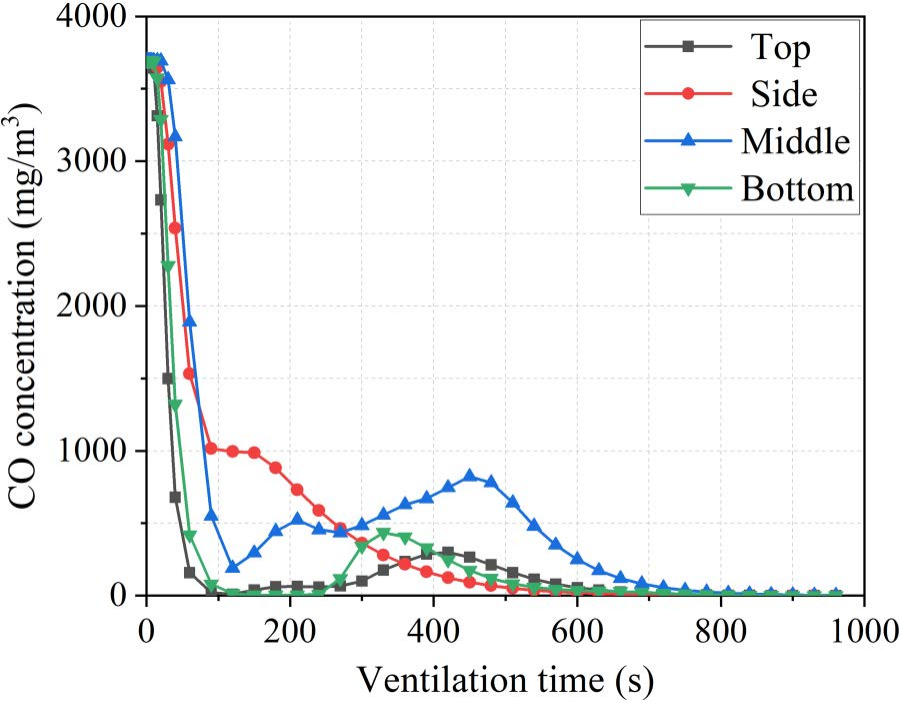
CO concentration variation process with different duct positions.

**Fig 10:**
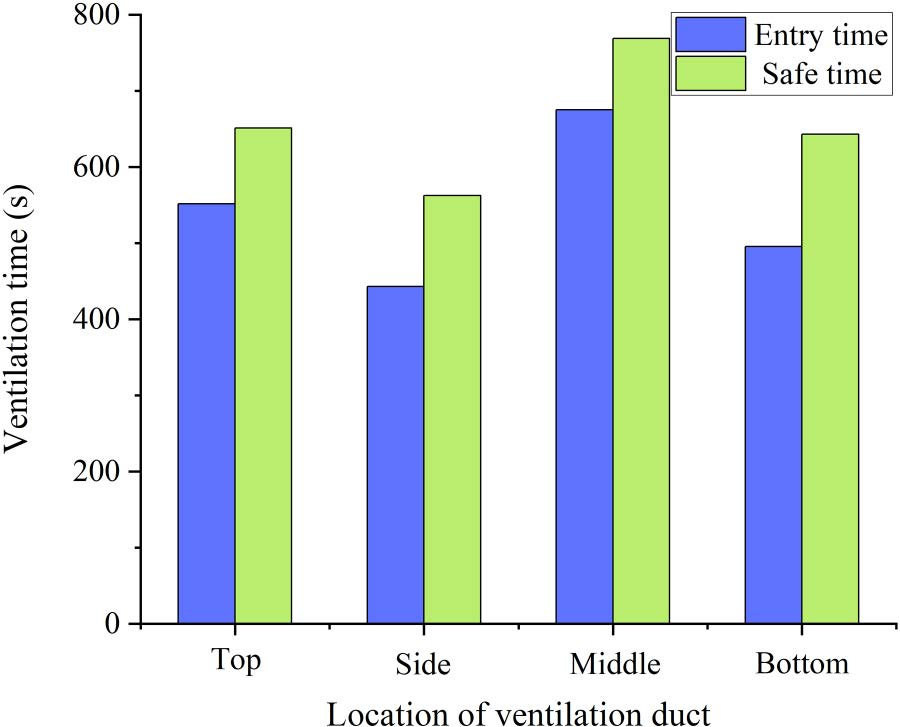
Ventilation time required with different duct positions.

**Fig 11:**
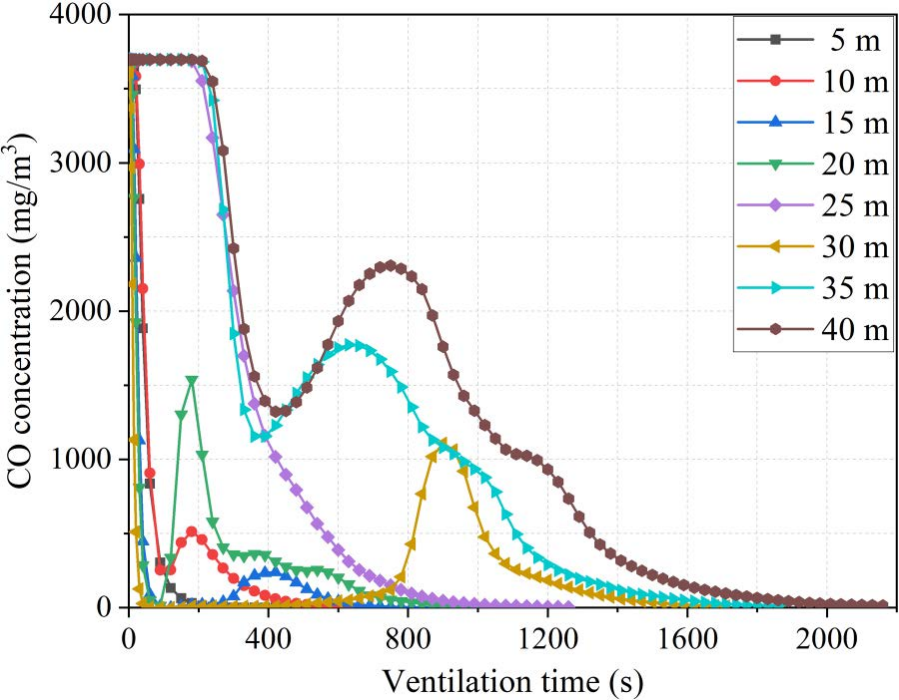
CO concentration variation process with different distance between duct outlet and working face.

**Fig 12:**
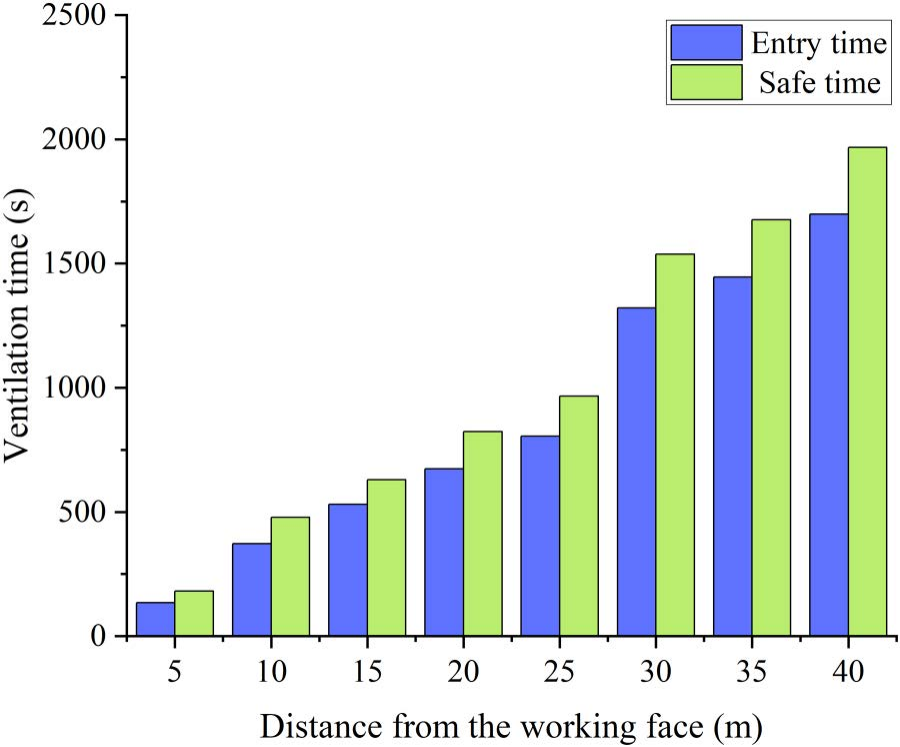
Ventilation time required with different distance between duct outlet and working face.

**Fig 13:**
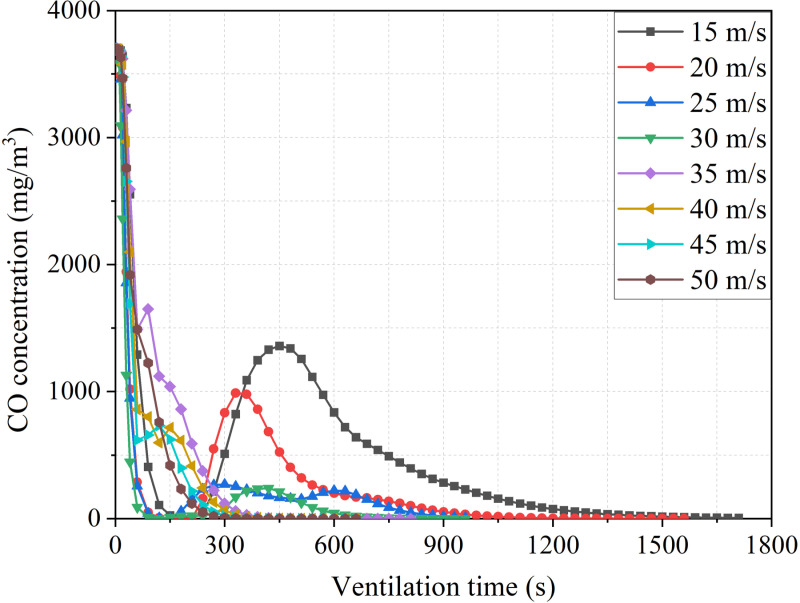
CO concentration variation process with different duct velocity.

**Fig 14:**
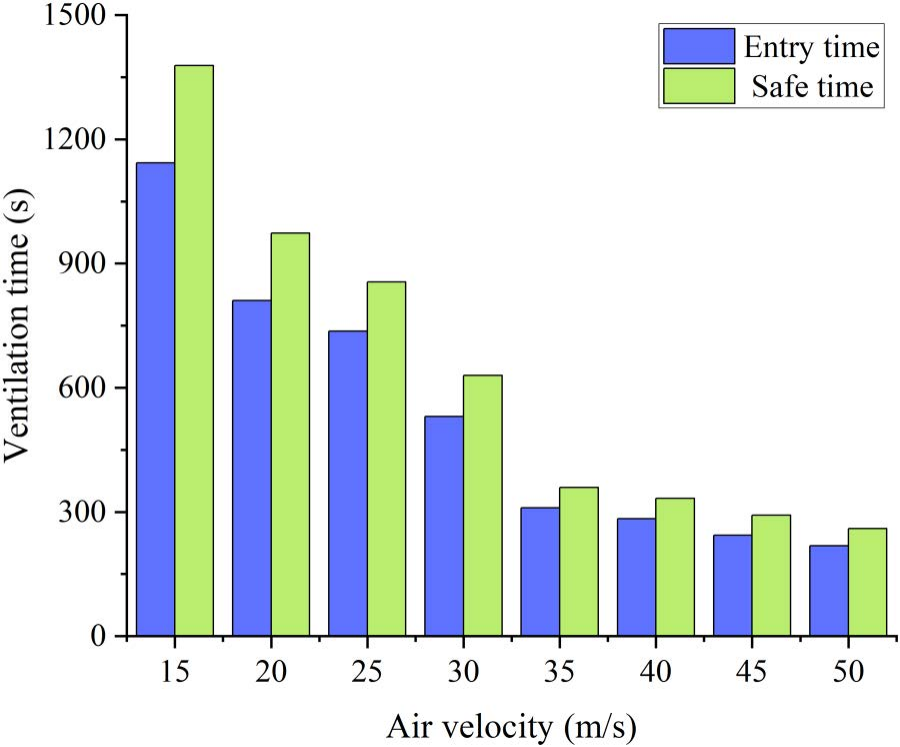
Ventilation time required with different duct velocity.

Fig 9–14-14 show the CO gas change process in the stagnant zone inside the tunnel, as well as the time required for the tunnel to reach concentration standards of 100mg/m^3^ and 30mg/m^3^. Under different conditions, the concentration of pollutants in the stagnant zone shows a pattern of first decreasing and then increasing. As ventilation continues, the concentration gradually decreases until it is eliminated. The reason for this phenomenon is that the concentration of pollutants in the stagnant zone is diluted with the addition of fresh air at the beginning of ventilation. As fresh air continues to flow in, pollutants in the remaining zone of the tunnel gradually move towards the duct outlet. The transport velocity of pollutants in the stagnant zone slows down due to the influence of vortex. Therefore, the concentration of pollutants shows a pattern of small-scale increase. Subsequently, the pollutants in the stagnant zone gradually diluted and decreased with the continuous addition of fresh air.

It can be seen that the pollutant removal process in the stagnant zone is smoother when the duct is arranged on the side wall of the tunnel from Fig 10. And the time required to reduce the concentration of pollutants in the tunnel to the safe concentration standard is also the shortest. On the contrary, the concentration in the stagnant zone undergoes two increases process when the duct is arranged in the middle of the tunnel. And the ventilation time required to reduce the concentration of pollutants to the safe concentration standard is also the longest. Therefore, the arrangement of air ducts on the side of the tunnel is the easiest way for pollutants to move out of the stagnant zone, and it is also the most convenient solution for pollutants to be extracted from the tunnel under extracted ventilation.

The different distances between the duct outlet and the working face result in two types of changes in pollutant concentration in the stagnant zone, as shown in Fig 11. When the distance is less than 20m, the concentration of pollutants shows a significant decrease at the beginning of ventilation. Subsequently, the concentration of pollutants showed varying degrees of increase. Finally, the concentration of pollutants decreases until they are eliminated. When the distance is greater than 25m, there is no significant change in the concentration of pollutants in the stagnant zone within 200s of ventilation. As the ventilation time continues to increase, the concentration in the stagnant zone shows a pattern of first decreasing, then increasing, and then decreasing again. The reason for this phenomenon is that the distribution pattern of the stagnation zone varies due to the different distances between the duct outlet and the working face. The distribution range of the stagnation zone gradually expands as the distance between the duct outlet and the working face increases. During the initial period of ventilation, fresh airflow is insufficient to reach the flow rate that dilutes the concentration of pollutants in the stagnant zone.

It can be seen that the low air velocity causes more complicated removal of pollutant concentrations in the stagnant zone from Fig 12. The increase in air velocity leads to a smaller increase in pollutant concentration. Meanwhile, the shorter the ventilation time required for pollutants in the stagnant zone to reach a safe concentration standard. The dilution rate of pollutant concentration by fresh air slows down due to the decrease in air velocity. Subsequently, pollutants from the remaining zone of the tunnel slowly entered the stagnant zone. Due to the slow dilution effect, the concentration in the stagnant zone increased significantly again.

Through the comparison of ventilation time, it was found that when the air velocity is greater than 35m/s, the trend of decreasing ventilation time gradually slows down. Therefore, it is more reasonable to control the air velocity at around 35m/s. In addition, when the distance between the duct outlet and the working face is greater than 25m, there is a significant increase in ventilation time. It is reasonable to control the distance at around 25m.

Based on the numerical simulation results, a comparison of ventilation efficiency under different parameters can be obtained. The ventilation time required for the tunnel to reach the safety concentration standard needs to increase by 140% for every 5m increase in the distance between the air duct outlet and the working face. The ventilation time required for the tunnel to reach the safe concentration standard increases by 62% for every 5m/s increase in air velocity of the ventilation duct.

In order to verify the rationality of parameter settings, this study adopted an improved Technique for Order Preference by Similarity to an Ideal Solution (TOPSIS) method for result comparison. The average velocity Vy = 0 in the y = 0 plane (longitudinal section of the tunnel center), the average velocity Vz = 1.6 in the z = 1.6m plane (human respiratory height plane), the average velocity Vx = 0 in the x = 0 plane (working face of the construction tunnel), and the overall average velocity V of the tunnel are used as dependent variable indicators to analyze the characteristics of the flow field under various working conditions. The orthogonal experimental scheme and numerical calculation results are shown in the Table 1.

**Table 1:** Comparison of simulation results for various operating conditions.

Working conditions	Experimental factors			Average flow velocity in stable section of tunnel (m/s)	Time for tunnel pollution to reach the standard (min)	Final score calculated by TOPSIS method
	Distance between the duct outlet and working face (m)	Air velocity (m/s)	Air duct location			
1	10	20	Top	1.078	16.23	0.0472
2	10	30	Side	0.990	10.11	0.0873
3	10	40	Middle	1.044	7.33	0.0903
4	20	20	Top	1.081	15.22	0.0371
5	20	30	Side	1.078	9.57	0.0627
6	20	40	Middle	1.062	6.78	0.1368
7	30	20	Top	0.746	18.21	0.0067
8	30	30	Side	1.078	13.04	0.0573
9	30	40	Middle	1.396	7.98	0.0926

**Fig 15:**
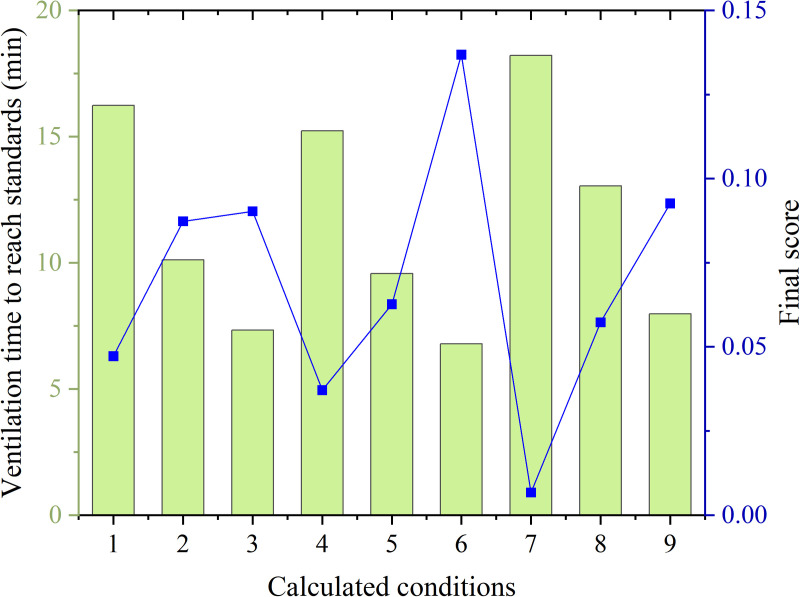
Results of different calculated conditions.

To avoid the limitations of obtaining evaluation results based on the improved TOPSIS theory and to optimize the design scheme more scientifically, an analysis and comparison of the ventilation flow field, distribution of harmful gas inside the tunnel were conducted for all established working conditions in Fig 15. The rationality of parameter configuration is judged based on two indicators: the average flow velocity in the stable flow section of the tunnel and the reduction of harmful gas concentration to the safe concentration standard. The results indicate that the construction arrangement for working condition 4 is more reasonable. Parameter settings not only improve the flow field distribution and vortex zone, but also enable the discharge of harmful gases in a shorter period of time, which can better meet the requirements of construction ventilation safety. Therefore, the theoretical derivation results and simulation results have been well validated.

### 3.3 Study on pollutant transport mechanism during extracted ventilation

**Fig 16:**
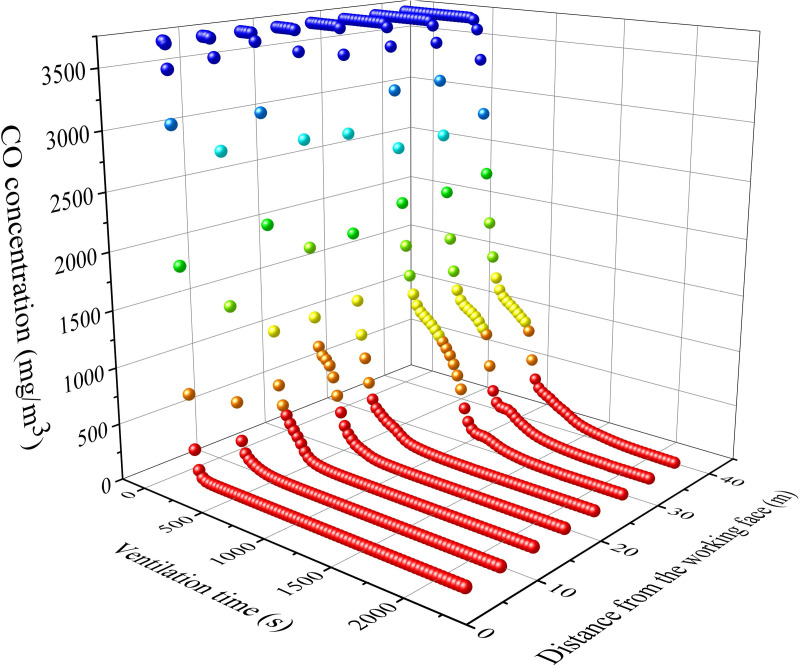
CO concentration variation at the air duct outlet with different distance between air duct outlet and working face.

**Fig 17:**
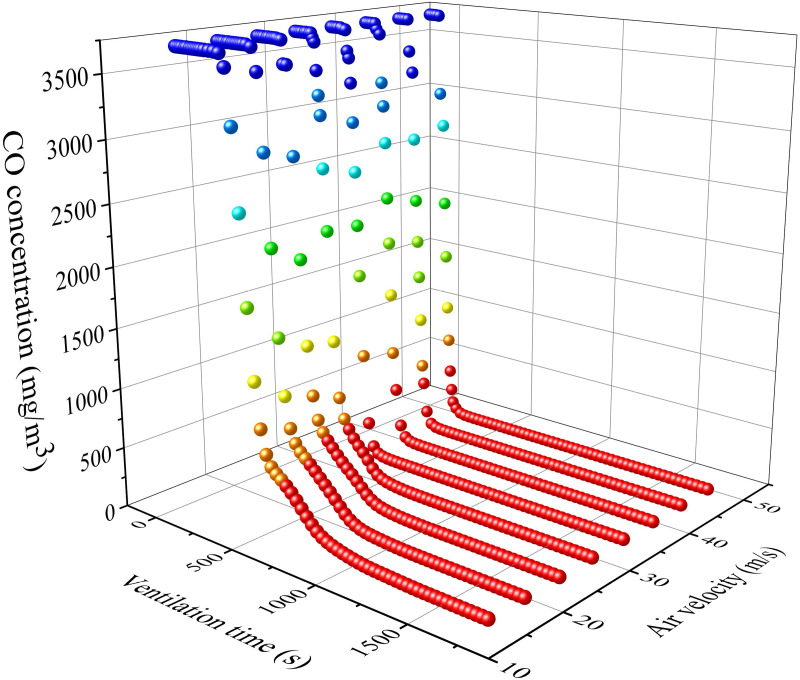
CO concentration variation at the air duct outlet with different air velocity.

The most important task of ventilation in tunnels constructed using drilling and blasting method is to remove pollutants from the tunnel. According to pollutant transport laws, the distribution of pollutants inside the tunnel is inhomogeneous. The distribution of stagnant zone of pollutants is difficult to predict and control under different ventilation conditions. The pollutants extracted from the air duct can be used as the object to calculate the residual harmful gases inside the tunnel. The calculation was carried out using the distance between the air duct outlet and the working face and the air volume as variables. The concentration change process of harmful gases at the air duct outlet is shown in Figs 16-17.

From Figs 16-17, it can be seen that the changes in pollutant concentration are mainly divided into two processes: extraction and dilution. In the early stage of ventilation, the CO concentration rapidly decreases with time. The polluted gases inside the tunnel are mainly discharged through the air duct. The emission of pollutants is mainly extracted during this stage. As the ventilation time gradually increases, the decrease in harmful gas concentration gradually tends to flatten after the concentration inflection point (about 5 minutes of ventilation). The emission of pollutants is mainly due to dilution during this stage.

The slope after the curve turning point increases with the distance between the air duct outlet and the working surface. This indicates that the dilution process slows down with increasing distance. The slope before the curve turning point increases with the increase of air velocity.The efficiency of extracting pollutants from the air duct increases with air velocity. Therefore, the dilution effect of pollutants in the stagnant zone is inversely proportional to the distance between the air duct and the working face. The extraction of pollutants is directly proportional to the air volume of the fan.

The mass of pollutants extracted from the air duct is obtained by integrating the CO concentration change curve at the duct outlet. The results are shown in Table 2.

**Table 2:** Calculation results of integrated concentration of polluted gases at the duct outlet.

Distance between air duct outlet and working face (m)	Volume of blasting throwing area (m^3^)	Fitting function expression (mg)	Integral result (kg)	Correlation coefficient	Error(%)
5	26.145	C_d_ = 9227.1exp^-0.023t^	104.687	0.9967	0.8
10	29.525	C_d_ = 9120.1exp^-0.016t^	111.337	0.9458	2.3
15	32.904	C_d_ = 21258exp^-0.013t^	130.515	0.9834	1.7
20	36.283	C_d_ = 45638exp^-0.011t^	134.895	0.8117	5.3
25	39.663	C_d_ = 56091exp^-0.008t^	152.735	0.9483	2.2
30	43.043	C_d_ = 66524exp^-0.006t^	177.294	0.9441	2.2
35	46.422	C_d_ = 84270exp^-0.005t^	177.054	0.8656	4.6
40	49.802	C_d_ = 100683exp^-0.004t^	200.259	0.9944	0.9
Air velocity (m/s)	Volume of blasting throwing area (m^3^)	Fitting function expression (mg)	Integral result (kg)	Correlation coefficient	Error(%)
15	32.904	C_d_ = 13575exp^-0.006t^	120.464	0.9964	0.8
20	32.904	C_d_ = 16147exp^-0.009t^	133.818	0.9858	1.2
25	32.904	C_d_ = 18369exp^-0.009t^	133.965	0.9207	2.3
30	32.904	C_d_ = 21258exp^-0.013t^	130.515	0.9834	1.7
35	32.904	C_d_ = 30110exp^-0.020t^	123.917	0.9723	3.1
40	32.904	C_d_ = 33673exp^-0.029t^	130.112	0.9716	3.3
45	32.904	C_d_ = 38340exp^-0.035t^	128.322	0.9685	1.8
50	32.904	C_d_ = 46367exp^-0.037t^	128.325	0.975	1.5

According to the calculation results in the table, the concentration change of pollutants at the air duct outlet can be fitted with an exponential function. The fitting of the function integral is basically consistent with the initial applied mass of CO gas. The reason for the error is the retention effect of the vortex zone on harmful gases. The convection and mixing of airflow under different conditions lead to the retention of pollutants in the tunnel. The fitting error under multiple conditions is within the allowable range. The results have good feasibility for further research.

The evolution of CO concentration at the cross-section of the duct can be expressed as:


$$C_{d}=a\exp^{bt}$$ (11)

**Fig 18:**
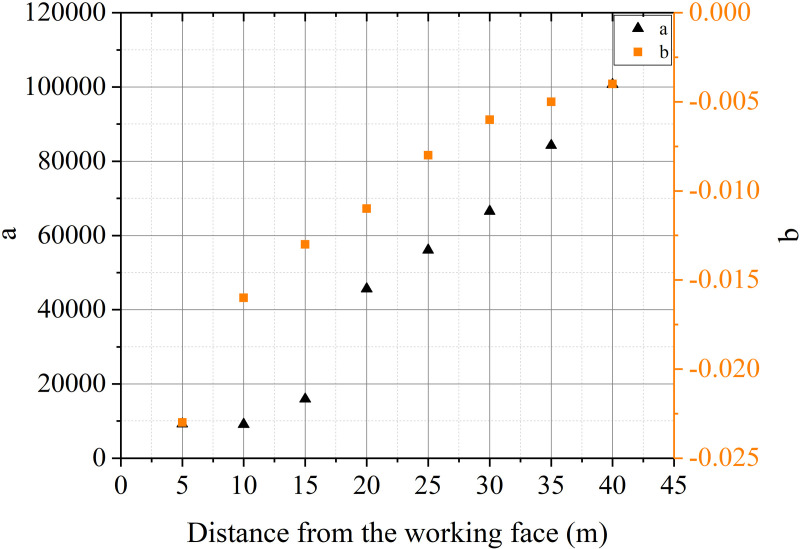
Relationship between C_d_ and t with different distance between air duct outlet and working face.

**Fig 19:**
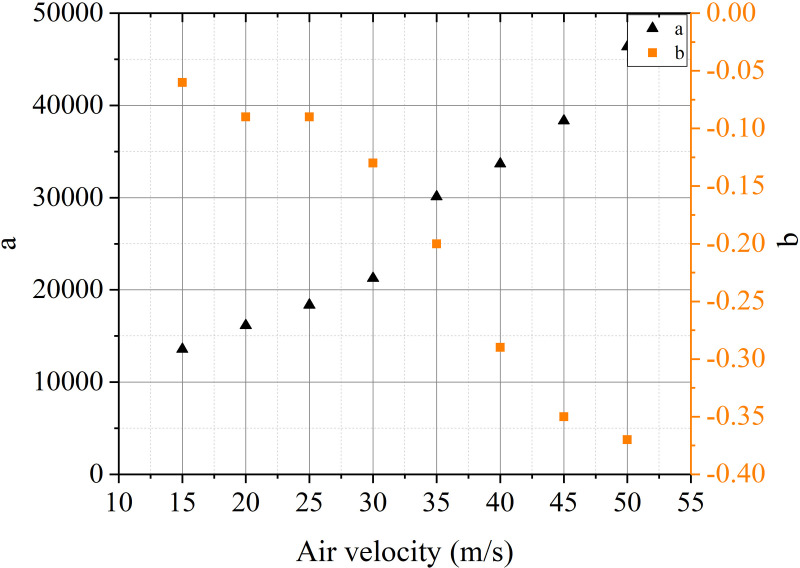
Relationship between C_d_ and t with different air velocity.

Figs 18-19 indicate that the attenuation of CO concentration at the duct outlet follows the variation law of the e-exponential function. Based on the laws of mathematical statistics, the function expression is shown in Eq. (11). The coefficient a is generally positively correlated with the distance of the duct outlet and the air velocity. The coefficient b is roughly positively correlated with the distance of the duct outlet and negatively correlated with the air velocity.

### 3.4 Derivation of ppone.0322984ollutant transport equation during extracted ventilation

Similar to the forced ventilation tunnel, the control body is also defined in the extraction ventilation tunnel [18–19]. A new correlation for calculating the required air supply volume can be obtained considering the dilution and convective diffusion of pollutants.

Pollutants quickly spread during blasting. The convective mixing effect between pollutants and air is strong. The dilution of CO gas mainly occurs within the control body. The dilution volume is $V_{I}=A\times L_{0}$, where A is the cross-sectional area of the tunnel; $L_{0}$ is the length of control body.

The ventilation time t is divided into n time periods, Δt=t/n. The fresh air entering the control body after time Δt is Q×Δt. At the same time, dirty air with a Q×Δt concentration of $C_{d}$ is discharged from the control body through the air duct outlet. Assuming the initial concentration of harmful gases in the control body is $C_{\text{0}}$, the dilution equation for pollutant gases in an extracted ventilation tunnel can be described as:


$$C=\underset{n\to \infty }{\lim}\;C_{n}=\underset{n\to \infty }{\lim}\;C_{0}{\left(V_{I}/\left(V_{I}+Q\Delta t\right)\right)}^{n}=\underset{\Delta t\to 0}{\lim}\;C_{0}{\left(V_{I}/\left(V_{I}+Q\Delta t\right)\right)}^{t/\Delta t}=C_{0}\exp^{-Qt/V_{I}}$$ (12)

where Q is the ventilation rate, m^3^/min; t is the ventilation time, min; $V_{I}$ is the volume of control body, m^3^; C is the average CO concentration at t min.

In existing studies [16,18,19], it is assumed that fresh airflow enters the control body and is uniformly mixed with pollutant gases. The study is based on the average concentration at each time. According to the CO migration law observed by the model results, polluted gases are not uniformly distributed within the control body. The concentration of pollutants at the air duct outlet is $C_{d}=f(t)$. After time Δt, the mass of harmful gases extracted from the air duct:


$$M_{d}=C_{d}\times v\times s\times \Delta t$$ (13)

The average concentration of harmful gases in the control body is:


$$C=(C_{0}\times V_{I}-C_{d}\times v\times s\times \Delta t)/V_{I}=C_{0}\text{-}C_{d}\times v\times s\times \Delta t/V_{I}=C_{0}\text{-}C_{d}\Delta Qt/V_{I}$$ (14)

The dilution equation inside the extracted ventilation tunnel can also be described as:


$$C=\underset{n\to \infty }{\lim}\;C_{n}=C_{0}-C_{d}Qt/V_{I}$$ (15)

The method of Least-squares [19,36] can be used to the CO concentration evolution. Eq.(11) can be derived as:


$$C/C_{0}=c{\left(Qt/V_{I}\right)}^{d}$$ (16)

By combining Eq.(14) and (15), the error caused by the uneven spatial distribution of CO gas can be eliminated.


$$\text{1}-C_{d}Qt/(C_{0}V_{I})=c{\left(Qt/V_{I}\right)}^{d}$$ (17)

Eq. (16) can be further written as:


$$\text{In(}C_{0}V_{I}-C_{d}Qt)=Ine+fIn\left(C_{0}Qt\right)$$ (18)

**Fig 20:**
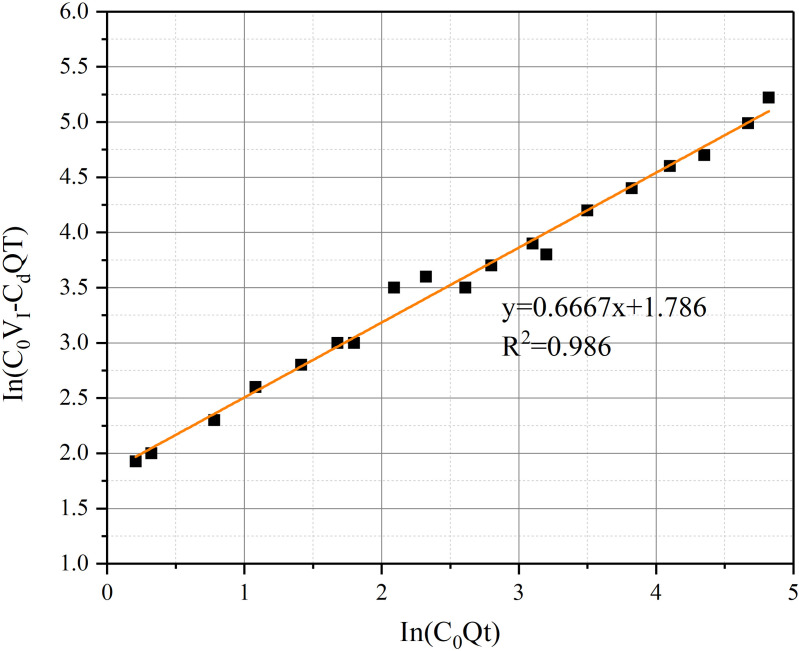
Relationship between In(C_0_V_I_-C_d_Qt)C_d_ and In(C_0_Qt).


Taking the distance between the air duct outlet and the working face as 15 m and the air velocity of 30 m/s as an example, according to Fig 20, Eq.(17) can be represented as:

$$\text{In(}C_{0}V_{I}-C_{d}Qt)=\text{0.6667}In\left(C_{0}Qt\right)+\text{1.786}$$ (19)

According to the Code for Design of Railway Tunnel [35], the initial CO concentration at the working face is:


$$C_{\text{0}}=Gb/AL$$ (20)

where G is the amount of explosive used for blasting, kg; b is the amount of harmful gas produced by unit mass explosives, m^3^/kg; L is the blasting throw distance, m; A is the cross-sectional area of the tunnel, m^2^.

When the concentration $C_{d}$ at the air duct outlet is 0mg/m^3^, it can be concluded that the pollutants inside the tunnel meet the safety concentration standard. Substituting Eq. (17) and (20) into Eq. (18), Q can be further expressed as:


$$Q=\text{0.7513}AL\sqrt{Gb}/t$$ (21)

The results are similar to the Voronin equation [35], but the calculations are more precise. The calculation formula as presented in Eq. (21) is suitable for estimating the air volume of the extracted ventilation fan used in tunnel working faces using blasting construction methods. When selecting the type of ventilation fan during the construction preparation stage, actual parameters can be used to fit an estimation function, which can be used as a calculation formula for the required air volume to discharge blasting smoke.

Two engineering cases were used for formula calculation comparison. Based on the theoretical foundation used above, fitting formulas for required air volume in different engineering cases were derived based on actual parameters. The calculation results are shown in Table 3.

**Table 3:** Engineering parameters and air volume determined for the case tunnels.

Parameter	Lianghekou Tunnel	Thoreau River diversion tunnel
Tunnel length (km)	6	6
Cross-sectional area of tunnel (m^2^)	67	18
Cross-sectional shape	City gate shape	City gate shape
Explosive dosage (kg)	60	210
Ventilation time (min)	30	30
Distance between air duct outlet and working face (m)	27	35
Air volume obtained by voronin equation (m^3^/min)	4791.77	468.85
Air volume obtained by fitting formula (m^3^/min)	4845.67	505.33
Error (%)	1.12	7.78

According to the calculation results in Table 3, it is found that the fitting results based on this study are similar to those calculated by Voronin’s formula. It proves the feasibility of deriving the theory.

The formula based on numerical simulation considers the special mechanism of pollutant stagnation zone. The formula integrates more practical ventilation factors in tunnels, such as the complex airflow, the distribution and diffusion of harmful gases in stagnant zone, and so on. The fitting formula is more in line with the actual situation of the specific project. The Voronin formula is based on some simplified assumptions and empirical summaries, mainly considering the basic parameters of tunnels (such as tunnel cross-sectional area, number of boreholes, explosive dosage, etc.) and ventilation time, which has certain universality and adaptability. After using two formulas for calculation, the calculated ventilation air demand results are compared. If the calculation results differ significantly, it may be due to the special nature of the project, which leads to an increase in ventilation demand in the stagnant zone inside the tunnel. The Voronin formula cannot accurately consider the influence of stagnation zones. In practical applications, it is necessary to further revise and optimize the formula based on on-site monitoring data to improve its accuracy and reliability. By continuously monitoring and providing feedback, the level of ventilation design and the safety of the project can be improved. With the continuous development of ventilation technology and changes in engineering requirements, it is necessary to update and improve formulas in a timely manner to adapt to new technological and engineering needs.

## 4. Conclusion

This study conducted an in-depth exploration of the characteristics of the airflow field and the migration laws of harmful gases within the ventilation tunnel during the construction period of a single-head excavation tunnel, based on a simulation model of the tunnel ventilation system. The main conclusions drawn are as follows:

(1) Under the conditions of extracted ventilation, the instability and turbulence effects of the fluid jointly result in a vortex zone near the tunnel working face. The swirling effect, vortex zone, and low-velocity zone are the primary factors leading to the formation of a pollutant stagnation zone. The concentration of pollutants in the stagnation zone exhibits a pattern of first decreasing, then increasing, and finally decreasing with the duration of ventilation.
(2) The emission process of pollutants is primarily divided into extraction and dilution phases. The dilution effect of pollutants in the retention zone is inversely proportional to the distance between the air duct and the working face. The extraction of pollutants is directly proportional to the airflow of the fan. Specifically, for every 5-meter increase in the distance between the air duct outlet and the working face, the ventilation time required for the tunnel to reach the safety concentration standard increases by 140%. Similarly, for every 5 m/s increase in the air velocity of the ventilation duct, the required ventilation time increases by 62%. Furthermore, the attenuation of pollutant concentration at the duct outlet follows the variation law of the exponential function, with the coefficients of this function linearly related to distance and air velocity.
(3) Employing an improved TOPSIS method, the study evaluated the calculation results of established operating conditions. The parameter settings selected through this method not only optimize the flow field distribution and area of the vortex zone but also enable the emission of harmful gases within a shorter time frame, effectively enhancing construction ventilation safety.
(4) Through parameter inversion and numerical integration, the study derived an empirical calculation expression for air supply volume, fully considering the effects of airflow convection and diffusion. This derivation method greatly reduces calculation errors. During the construction preparation phase, actual parameters can be used to fit the estimation function, providing a scientific basis for the selection of ventilation fans. This estimation function has broad applicability to the same type of extracted ventilation tunnel and offers significant reference value.
